# Leadership of AAAP scientists and journals in animal science: achievements, limitations, and challenges

**DOI:** 10.5713/ab.22.0409

**Published:** 2022-11-14

**Authors:** Jong K. Ha

**Affiliations:** 1 Animal Bioscience, Seoul 08776, Korea

**Keywords:** AAAP, AJAS, Animal Bioscience, Asian Animal Science

## Abstract

The Asian-Australasian Association of Animal Production Societies (AAAP), the only international scientific organization in animal science representing Asia and Pacific region, showed a remarkable development since its inception in 1980. The number of member countries increased from 8 to 19 while 19 congresses were held in 11 different member countries. The AAAP also helped creating the official journal, Asian-Australasian Journal of Animal Sciences in 1988 with the title being changed to Animal Bioscience in 2021. It is an open access journal indexed by most global databases and has become one of the most respected global journals in animal science. Through scientific meetings and journal publication, the AAAP has made a tremendous contribution to the advancement of animal science and industry throughout its member countries over the last 40 years. This paper summarizes the advances in animal science in the Asia–Pacific region, together with the roles of the AAAP scientists and journals in developing animal science.

## ADVANCES IN ANIMAL SCIENCE IN ASIA AND THE PACIFIC REGIONS

The AAAP region covers diverse environments, religions, cultures, economies, and nature. Asia alone harbors more than 50% of the global population [1] that is directly linked to food supply. Asia produces over 60% of the global foods, including livestock products [2]. Additionally, by 2050, more than half of the global demand and production are expected to come from this continent. More importantly, the Asia–Pacific region has become a leading center of science and technology development.

This might be a good opportunity to examine the contributions of AAAP countries to the advancement of science, especially animal science.

Reports on scholarly activities over a long period on a global scale are not easy to obtain because of limited availability of realistic data. A recent study showed interesting data illustrating the trend of global scholarly activities. This study used the Scopus database to examine the publication trends of scholarly journal articles, including the science of 12 major countries, during the last 120 years [3].

**Figure 1:**
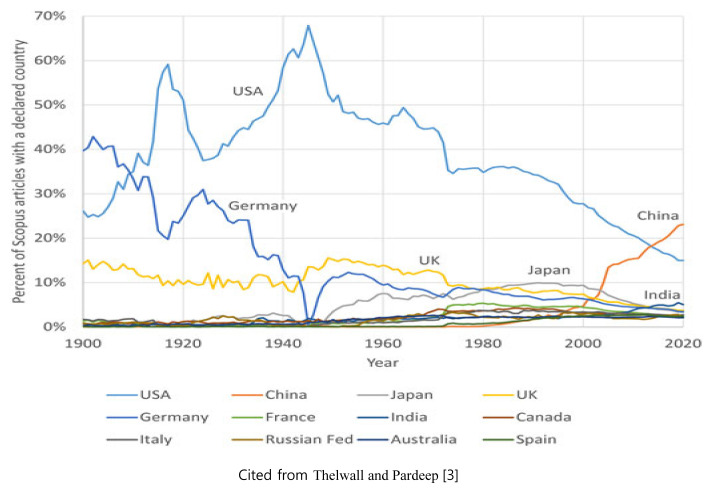
The percentage of Scopus articles with first author from the 12 countries with the most articles.

As depicted in Figure 1, in 1900, over two-thirds of the first authors of scholarly articles indexed by Scopus were from Western countries such as the USA, Germany, and the UK, dominating publication activities for most of the time during the last 120 years. However, by 2020, China produced more articles than the USA and Germany combined, and India published the third most articles, clearly demonstrating that scholarly activities in the AAAP region have recently become stronger. This is just one example of the transfer of scholarly activities from the Western world to the AAAP region.

**Figure 2:**
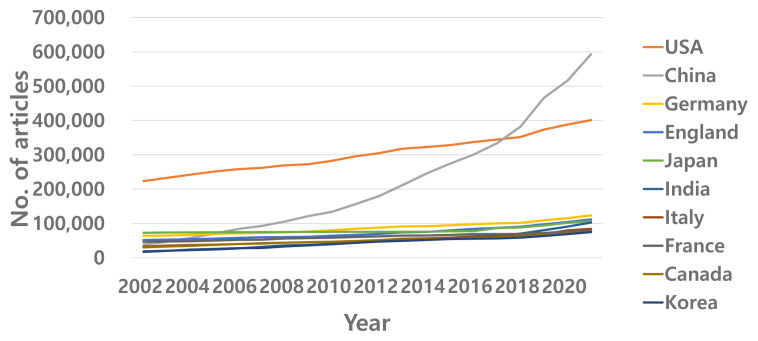
Annual growth rate of research output of top 10 countries: article only (overall average 0.058/yr; China average 0.155/yr).

**Figure 3:**
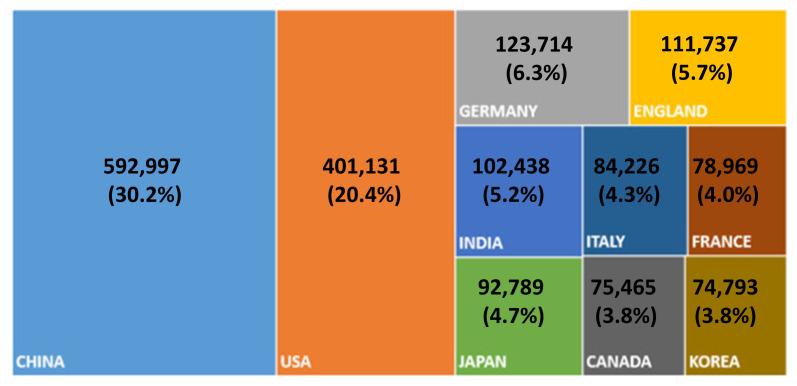
SCIE journal articles published by top 10 countries in 2021 (All science sectors).

This is clearer if one takes a look at the publication trend in more recent years using different databases [4]. The overall global publication activity continuously increased by approximately 6% annually during the period between 2002 and 2020. Contrastingly, China moved forward at a phenomenal speed of almost a 16% annual increase during the same period (Figure 2). Therefore, China has become a front-runner in R&D activities in science. According to the most recent JCR report [4], China produced almost one-third of global SCIE articles. Other AAAP member countries, including India, Japan, and Korea, are also strong in scientific research, as shown in Figure 3.

**Figure 4:**
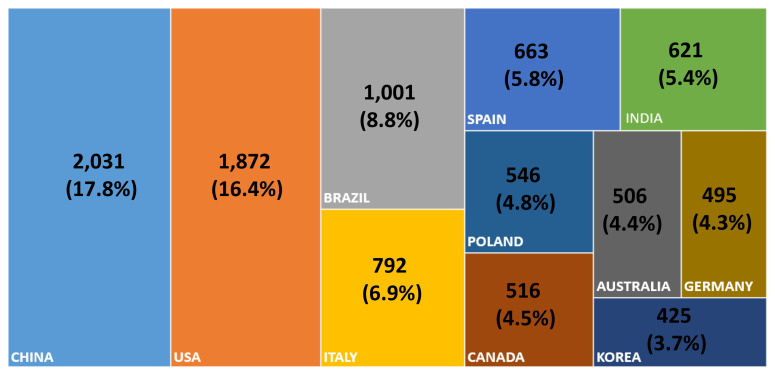
SCIE journal articles published by top 11 countries in 2021 (Ag, Dairy & Anim Sci).

If one narrows down the scope from all disciplines of science to animal science, the same trend can be seen in which the AAAP scientists in animal science in 2021 show outstanding performance in terms of the output of research articles (Figure 4). Four AAAP countries (i.e., China, India, Australia, and Korea) produced over 30% of the global research output in agriculture, dairy, and animal science. These data clearly indicate that the AAAP countries lead in global animal science research.

Considerable improvements have been made in journal publication activities by the AAAP member countries. Currently, 63 journals in the animal science category are indexed by the Science Citation Index Expanded (SCIE) [4]. Of these, 13 journals (21%) were published by the AAAP member countries (Table 1). The proportion of SCIE journals in animal science published by the AAAP countries was 23% of the global total in 1980, indicating that no major changes in the proportion have been made over the last 40 years. However, there is a good chance to have more SCIE journals published by the AAAP countries in the near future because some journals of the AAAP member countries have already entered the Emerging Sources Citation Index (ESCI) category, which is a type of candidate journal that has the potential to be promoted to the SCIE category. To date, over 40% of the global ESCI journals have been published in the AAAP region.

**Figure 5:**
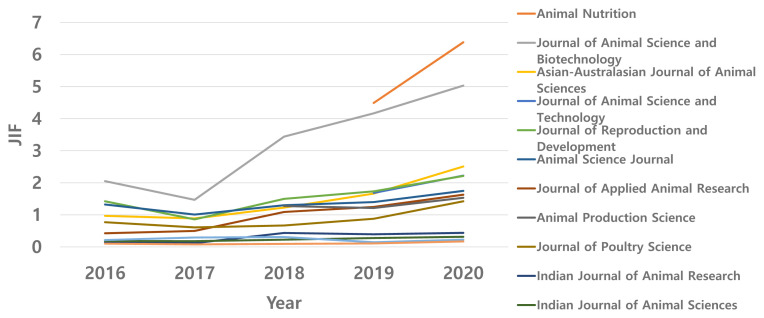
Change of impact factor of 13 SCIE journals published in AAAP region (Ag, Dairy & Anim Sci).

The impact factor (IF) is one way of measuring the citation frequency of journals and is often considered as the degree of quality or reputation of journals. The IF of AAAP journals, similar to that of many other international animal science journals, has continuously improved in recent years. However, the magnitude of improvement within the AAAP varied considerably, as shown in Figure 5. The extraordinary performance of two recently established Chinese journals (Journal of Animal Science and Biotechnology and Animal Nutrition) was the main factor in boosting the average IF of AAAP journals, as clearly depicted in the figure. We congratulate the Journal of Animal Science and Biotechnology and Animal Nutrition for this feat.

**Figure 6:**
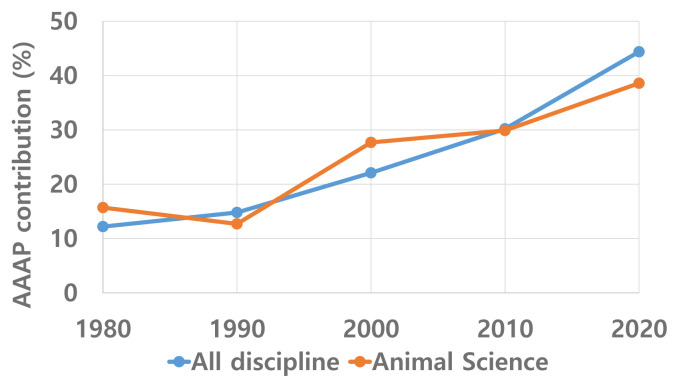
AAAP contribution to global research output in SCIE article publication (1980–2020).

It would be interesting to determine the contribution of AAAP to global research activity in animal science. When the AAAP was founded 40 years ago in 1980, its contribution to global research output in animal science was approximately 15% (Figure 6). Presently, it has increased to almost 40% of the global average, which is more than a 2.5-fold increase since 1980. This is a clear indicator of the increased share of Asia–Pacific contribution to global R&D activity. Therefore, the quantitative contribution of the AAAP countries to global animal science research activity has become much larger than before.

**Figure 7:**
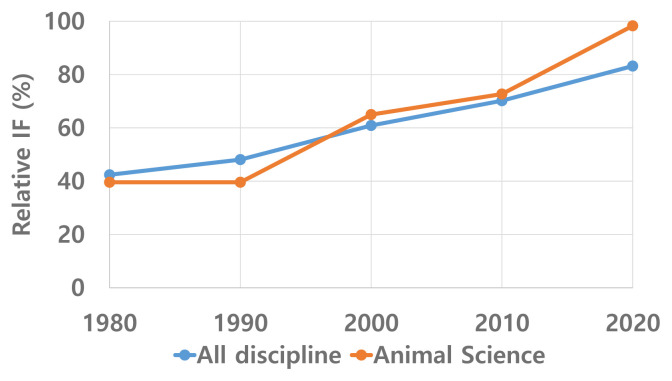
Competitiveness of SCIE journals produced in AAAP by impact factor (1980–2020).

We can see the same picture in journal article quality (Figure 7), although IF is not the only indicator of journal quality. The average IF of AAAP journals 40 years ago was only 40% of the global average, while it is essentially the same today.

Therefore, these data show that the R&D capability of the AAAP countries has improved substantially during the last 40 years since the birth of the AAAP. Of course, many factors, such as increased investment in research activity with economic growth, contributed to such results, but the roles of scientific organizations such as the AAAP cannot be ruled out for the development of animal science and the livestock industry.

## ROLES OF ASIAN-AUSTRALASIAN ASSOCIATION OF ANIMAL PRODUCTION SOCIETIES (AAAP)

Most scholarly organizations plan to hold conferences, congresses, or various scientific gatherings, and to publish journals to provide a proper platform for their members. It is considered that the AAAP has fully played the role of a scientific organization in both aspects during the last 40 years. In this section, I will summarize the major activities of the AAAP since its foundation in 1980.

The descriptions here are mostly based on official records of the AAAP Secretary General’s Office, together with previously published documents [5–8]. Personal communication with Prof. In K. Han, who had a huge influence on the creation of AAAP and its official journal, AJAS, has been a big input in the preparation of this review.

The 1st AAAP congress was held in 1980 under the leadership of the Malaysian Society of Animal Production. After eight years, the official journal AJAS was founded under the leadership of Prof. In K. Han, and later, the journal title was changed to Animal Bioscience (AB) in 2021. The permanent secretary general office was established in Korea in 2000, and different award programs were launched at 10-year intervals: 1990, 2000, and 2010.

The AAAP began with eight members and now has a total of 19 members, making it one of the most important international organizations in the world. Charter members were Australia, Indonesia, Japan, Korea, Malaysia, New Zealand, Philippines, and Thailand. Taiwan joined the association in the 2nd Congress held in the Philippines in 1982. Other countries were invited as members in different years, with China and Sri Lanka becoming the most recent AAAP members in 2006. Although the size of membership has more than doubled in the last 40 years, the list is not complete until many other Asian countries join our association. I hope the AAAP council continues to try inviting more nations as members so that other Asia–Pacific animal scientists can participate and benefit from our activities.

To date, 19 AAAP animal science congresses have been held in 11 different member countries (Table 2). Malaysia and Korea hosted thrice; Japan, Indonesia, Taiwan, and Thailand hosted twice; and Australia, New Zealand, India, Philippines, and Vietnam have also hosted the AAAP congress. Most congresses were held every two years except for a few cases (3rd, 9th, and 19th).

It should be emphasized that the AAAP Congress is an important event for not only scientists but also other professionals in the livestock industry. Thus, it is one of the most important scientific gatherings for many scientists to exchange ideas, disseminate animal science, and develop friendships and professionalism. These meetings, especially during the early days, were rare opportunities for scientists from many parts of Asia, especially young scientists, and in that aspect, we must appreciate the great contribution of the AAAP congresses. The participants were not limited to member countries, Asia, or the Pacific region. Most congresses were attended by both member and non-member countries with almost 30 different nationals, except for a few meetings, which shows that the congresses gained international importance and popularity.

One of the most important outcomes of the congress may be the proceedings that compile plenaries, symposiums, and scientific presentations, usually in abstract form. International meetings were used to distribute several volumes of heavy thick proceedings in a conference bag in the past, although an increasing number of organizers prefer to put conference contents into handy and easy-to-carry devices, such as disks and USB. Indeed, a few other congresses do not provide hard copies or conference contents in any form of device, but provide participants with access to proceedings only in an online format. Technical development, together with the impact of the pandemic, brought about changes in many gatherings and meetings.

Awards are a good stimulus promoting excellence in research and development, and it is fortunate that the AAAP has created several award programs for its members.

One of the main award programs is the AAAP Animal Science Award, established in 2000 with a generous donation from the Hans’ Animal Life Science Foundation in Korea. The foundation supported the award program until 2008; thereafter, the award was funded by the country hosting the Congress. Fifteen eminent scientists received awards until 2022.

Another award program, the Woogene B&G Award, was initiated in 2010, funded by Woogene B&G (Korea), and all members of the AAAP are thankful to the company for their generous financial support.

Our association also has other award programs, especially for young scientists, as follows. One is the travel grant, and young scientists are chosen as awardees to support and encourage their participation. A grant is usually supported by a host country. Another program is the Best Presenter Award, which is given to young scientists (usually students) who show excellent performance in oral or poster presentations.

It should be recognized that these awards have been supported by the Japanese Society of Animal Science, Korean Society of Animal Science & Technology, and Prof. LC Hsia, Taiwan. The contributions and efforts of the Japanese and Korean Societies and Prof. Hsia are greatly appreciated.

## CREATION AND ACHIEVEMENT OF ASIAN-AUSTRALASIAN JOURNAL OF ANIMAL SCIENCES (AJAS)/ANIMAL BIOSCIENCE (AB)

The official journal of AAAP, Asian-Australasian Journal of Animal Sciences (AJAS), was founded in 1988 in Korea. According to two previous presidents of the AAAP, Profs. In K. Han and S. Jalaludin, the idea of an official journal publication at the beginning was not well accepted; member countries did not have a real need for such a journal, and people did not have a firm belief in the success of such publication activities in Asia. However, a few dedicated frontier scientists led by Prof. In K. Han, the first editor-in-chief, were able to turn AJAS into one of the most respected global journals in the animal science category.

As expected, collecting the manuscripts in the early days proved to be difficult. Less than 50 manuscripts were submitted in the first year; however, this rose to almost 1,000 annually in some years. At present, authors from more than 50 countries have chosen AJAS to report their research work. China and Korea are the major contributing countries. Approximately 70% of submissions are from the AAAP countries, with the remaining 30% originating from the non-AAAP region (Table 3).

Generally, the number of published articles reflects the number of submissions. Papers from the two most submitting countries, China and Korea, account for the highest number of publications. Publications by non-member countries such as the USA, Brazil, and Italy are also gradually increasing.

**Figure 8:**
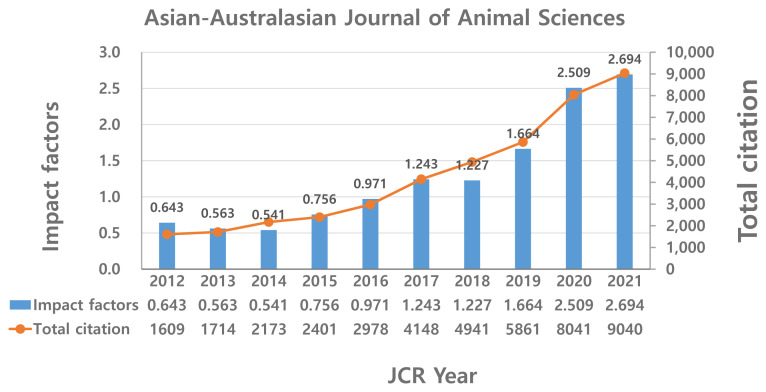
Trend in total citation frequency and two-year impact factor of AJAS (2012–2021).

The AJAS has made a tremendous improvement in citation frequency. When AJAS was indexed by the SCIE for the first time in 1997, the IF was lower than 0.1; however, it is now 2.7. The total number of citations in the first year was less than 100, but it was almost 9,000 in 2021, which is an approximately 100-fold increase over the past 20 years (Figure 8). It is expected that the IF of AB will likely decrease in the next two or three years due to the change in journal title from AJAS to AB; however, it will not influence the combined journal IF significantly and AB IF will return to the normal range.

For successful journal publication, continued and dedicated efforts by many researchers are required. In particular, small society journals such as AB cannot expect enough financial and manpower support from society, which requires dedication from many volunteers. Many volunteers are involved in journal publication. This is definitely a strong asset for AB.

Recent outstanding journal quality improvement was, to some extent, due to the recently completed 7-year development program (AJAS 2020 program). Table 4 summarizes some of the key achievements of this special program. Many innovative measures in editorial and journal management were adopted during the program period; consequently, journal visibility and citation frequency substantially improved. Total citation increased 4.7 times reflecting a similar level of increase in IF, which boosted the ranking from 37% to 78%, which is high enough to qualify as a Q1 journal.

Despite recent significant improvements in journal quality, continued innovation is essential to maintain as a leading journal in the ever-changing global publication environment. The AB should be able to provide the best content to become a highly prestigious and respected journal. Continued efforts are required to invite high-quality articles. Furthermore, AB should be able to provide a global standard system to stakeholders such as authors, reviewers, and the general public for efficient and easy access to AB systems.

The title of our official journal was changed from Asian-Australasian Journal of Animal Sciences (AJAS) to Animal Bioscience (AB) effective from January 2021. Through this change, it is anticipated that we can enhance journal brand identity, secure international leadership, and perhaps be able to cover the diverse interests of the animal industry and academia in the long run.

The AJAS also created an award program in 1990, which has been financially supported by Cargil AgriPurian Inc. (CAPI), Korea. During the early days, awardees were selected solely based on the excellence of research papers published by AAAP member scientists; however, in recent years, authors with the other qualifications such as contribution to journal development through citations and other services have also been chosen as awardees.

To date, 42 AAAP scientists have been recognized by this award program. Congratulations to all of these awardees, and their contribution and dedication to the advancement of the journal are greatly acknowledged. We also sincerely appreciate CAPI for their generous support since the inception of the award program.

## LIMITATIONS AND CHALLENGES FOR THE AAAP

Overall, it is needless to say that the AAAP played a key role in bringing animal scientists together and contributing to the development of livestock production in Asia and the Pacific. The AAAP never missed the regular Animal Science Congress, which served as an important forum for the exchange and dissemination of information and new ideas. Its official journal, AJAS, has become one of the major international journals in the field of animal science. In addition, the association has initiated various award programs to encourage research and other scientific activities of members.

Therefore, there is no doubt that the AAAP will continue to thrive, as was the case in the past. However, there are still some challenges and issues ahead of us, and it would be worthwhile to identify some of the key issues and propose possible approaches to dissolve them.

### Competitiveness

As is well known, many local, regional, and international associations and journals are similar in nature to the AAAP and AB. Therefore, the association and journal should be able to provide sufficient motivation to become our member, to attend our congresses, and to publish their work in our journal. Continued efforts to bring innovation to our association and journal will definitely be required to maintain competitiveness.

### Regional collaboration

If you look up proceedings and journals, you seldom see abstracts and articles produced from joint projects or collaborations with the AAAP members. More collaboration and interaction among the member countries is required. The AAAP members should seek more opportunities to provide their opinions on regional and global issues.

### International collaboration

We also need channels to establish more collaboration with other international organizations. This could be a simple exchange program for junior and senior scientists at various meetings. For instance, the mutual invitation of speakers to regular meetings can be attempted at the beginning. It can then be extended to more complicated forms of cooperation, such as co-publication or joint committee activities.

In addition to the AAAP, there are a few other international societies in the field of animal science. The best-known organizations are the World Association of Animal Production (WAAP), the European Federation of Animal Science (EAAP), the Latin American Association of Animal Production (ALPA), and the All Africa Society for Animal Production (AASAP). Of these, the EAAP is perhaps the most active association of a true international nature. It is active in expert exchanges, reciprocal session operations, and co-publications. It is also involved in the co-publication of Animal Frontiers, which publishes a type of position papers and is a joint venture between EAAP, American Society of Animal Science (ASAS), Canadian Society of Animal Science (CSAS), and American Meat Science Association (AMSA). The EAAP can be a model for the future development of the AAAP.

### Inequality among member countries

The member countries that sent the greatest number of delegates to the most recent five congresses have been the same in all five congresses: Japan, Korea, Thailand, Taiwan, and Indonesia (Table 5). More than 90% of the participants were from these five member countries.

The same member countries presented the most papers in the five congresses, as shown in Table 6.

In fact, those five countries plus China and India, published the most papers in AJAS, with little contribution from other member countries (Table 7).

Therefore, there is severe regional inequality in congress participation, research, and publication activities among our members. Of course, a prompt solution is not evident, but there must be some way to carry it out right away without any extra expenses or prior arrangement. For example, the host country of each congress may prioritize allocating travel grants to delegates from under-represented member countries or set aside at least the minimum number of speaker positions for these countries.

Inequality in publications is even more complicated. It not only involves publication cost (APC), but also plays a role in the level of research due to shortage or lack of grants and facilities, which cannot be solved in a short duration. International collaboration with established scientists may provide opportunities for them to upgrade their knowledge and skills necessary for more advanced research and to have a better chance of acceptance by the journal.

### Membership

There are 49 United Nations (UN) member countries in Asia. Of these, 16 Asian countries are AAAP members. Currently, our member countries are mostly located in northeastern and southeastern Asia. It is time to discuss the possibility of inviting Central and Western Asian countries as members. Of course, more efforts need to be made to complete the invitation to all northeastern and southeastern Asian countries.

### Constitution

The last, but not the least, limitation is the AAAP constitution. The AAAP statutes are simple and short documents, and yet they describe most of the basic rules necessary for the operation of the association. However, the document was enacted more than 40 years ago, and it surely deserves an in-depth review to determine if any revisions or updates are necessary.

I would like to propose the following 5 issues for future discussion.

*The AAAP membership* is granted to only one national society. Although this is probably the best policy, future discussion is needed to determine whether multiple organizations will be allowed. In addition, detailed and formal guidelines to evaluate their applications are required because currently no specific policy is set for this purpose other than submission of a simple letter of intention to the secretary of general.

*An annual fee* of 100 USD per membership was set in 1980. The reasonability of this current annual fees needs to be reviewed.

*Revenue* is one of the key constraints in the efficient operation of the association. The only revenue source is the annual fees, and more stable funding sources should be identified. It is hoped that the AAAP council considers the differential annual fee system and also reviews the possibility of imposing a levy of a certain percentage to congress registration fees, as proposed a few times in the past.

*The governance structure* of AAAP should be reviewed. This can be expanded to perform diverse activities. For example, we may need some standing committees (or ad hoc committees) to deal with the academic contents of congresses, international cooperation, some specific issues, etc.

*Congress sessions* have mostly been directed toward scientific activities. Of course, the main function of our congress is to provide a proper platform for scientists of the member countries, but simultaneously, the congress should be able to provide suitable programs for consumers, policymakers, and the general public. Thus, for instance, reach-out sessions for the layperson, youngsters, or children may be set up, and the sessions can be operated in the local language if necessary. This type of public lecture will become more important in the coming years because providing unbiased scientific information to the general public, especially to the younger generation, is one of the most efficient and secure ways to improve consumer perceptions of the livestock industry.

In summary, the AAAP has made splendid achievements during the last 42 years. Various records indicate that the AAAP has made tremendous contributions to the advancement of animal science and the animal industry in the Asia–Pacific region through scientific meetings and journal publications. With the Asia–Pacific region becoming a global leader in science and economy, it is expected that greater responsibility for the AAAP members is required for developing regional and global economies through animal science and the livestock industry. Continued innovations to the association and the journal are necessary to fulfill the aims of the AAAP.

## Figures and Tables

**Table 1:** Number of SCIE and ESCI journals in AAAP region in 2021 (Ag, Dairy, & Anim Sci)

Country	No. of SCIE	No. of ESCI	Total
India	4	3	7
Japan	3	-	3
China	2	-	2
Korea	2	-	2
Australia	1	-	1
Thailand	1	-	1
Iran	-	2	2
Bangladeshi	-	1	1
Indonesia	-	2	2
Total	13 (63)	8 (17)	21 (80)

**Table 2:** AAAP Animal Science Congress (1980–2022)

No	Country	Year	President	Organizing committee chairs	No of participant (country)
1	Malaysia	1980	S. Jalaudin	Osman B. Din	234 (15)
2	Philippines	1982	V.G. Arganosa	G.C. Orinion	777 (15)
3	Korea	1985	In Kyu Han	D.A. Kim	1,036 (28)
4	New Zealand	1987	A.R. Sykes	K.E. Jury	581 (28)
5	Taiwan	1990	T.P. Yeh	S.C. Chyr	659 (26)
6	Thailand	1992	C. Chantalakhana	C. Chantalakhana	887 (30)
7	Indonesia	1994	E. Soetirto	E. Soetirto	894 (31)
8	Japan	1996	T. Morich	S. Watanbe	1,110 (30)
9	Australia	2000	J. Ternouth	T. Scott	733 (31)
10	India	2002	P.N. Bhat	V.L. Chopra	471 (23)
11	Malaysia	2004	Z.A. Jelan	L.C. Liang	427 (27)
12	Korea	2006	I.K. Paik	S.J. Ohh	1,250 (32)
13	Vietnam	2008	Nguyen Van Thien	Nguyen Van Thien	1,273 (32)
14	Taiwan	2010	Liang Chou Hsia	Liang Chou Hsia	2,629 (34)
15	Thailand	2012	C. Kittayachaweng	Metha Wanapat	1,422 (42)
16	Indonesia	2014	Yudi Guntara	Budi Guntoro	1,174 (44)
17	Japan	2016	Seiichi Koizumi	Mitsuhiro Furuse	1,160 (27)
18	Malaysia	2018	Loh Teck Chwen	Loh Teck Chwen	646 (22)
19	Korea	2022	Yoo Yong Kim	Sejong Oh	1,446 (22)

**Table 3:** Manuscript submission to AJAS and AB by country in recent years

Country	2018	2019	2020	2021
China	330	339	306	230
Korea	116	148	103	81
India	58	53	46	33
Japan	33	21	13	12
Thailand	51	34	41	20
Brazil	62	70	56	28
Turkey	40	36	37	16
AAAP	707	718	614	450
Non-AAAP	282	281	260	131
Total	989	999	874	581

Submitted by 55 countries (16 AAAP and 39 non-AAAP countries).

**Table 4:** Key outcome of AJAS 2020 program

Areas	Measures and outcome
Visibility	Open access, XML, Facebook
Archiving	PubMed Central, DOAJ, DOI
Contents	Invited review, Special issues
Style & Format	Symbol, Cover, Referencing, General Guidelines
System	Homepage, Journal Management, Ethics
Total Citation	1,714 (2013)→ 5,861 (2019) → 8,041 (2020)
Impact factor	0.563 (2013) → 1.664 (2019) → 2.509 (2020)
IF Percentile	37% (2013) → 67% (2019) → 78.6% (2020)

The program was initiated in January 2014, ending in December 2020.

**Table 5:** Top 5 countries which sent the most number of participants to recent congresses

AAAP	Year	Venue	Countries	Top 5 / total	%
18	2018	Malaysia	MY, KR, JP, TW, ID (TH)	537/631	85.1
17	2016	Japan	JP, ID, TW, KR, TH	1,012/1,131	89.5
16	2014	Indonesia	ID, KR, JP, TH, TW	1,019/1,111	91.7
15	2012	Thailand	TH, KR, TW, JP, ID	876/1,010	86.7
14	2010	Taiwan	TW, TH, JP, KR, IR	2,494/2,601	95.9
Total/average			JP, KR, TH, TW (ID)	5,938/6,484	91.6

**Table 6:** Top 5 countries which presented the most number of papers in recent congresses

AAAP	Year	Venue	Countries	Top 5/total	%
18	2018	Malaysia	MY, KR, JP, TW, CN	322/529	60.9
17	2016	Japan	JP, ID, TW, KR, TH	725/834	86.9
16	2014	Indonesia	ID, KR, JP, TH, TW	643/725	88.7
15	2012	Thailand	TH, KR, TW, JP, IR	650/865	75.1
14	2010	Taiwan	TW, TH, JP, KR, IR	604/720	83.9
Total/average			JP, KR, TW (TH, ID, IR, CN)	2,944/3,673	80.2

**Table 7:** List of countries published the most articles in AJAS (1988–2021)

Member country	Articles published
Korea	1,693
China	931
Japan	744
India	545
Taiwan	239
Thailand	232
6/19 AAAP members	84%
